# Dr. L. B. Lester

**Published:** 1913-11

**Authors:** 


Dr. L. B. Lester, Geneva, 1864, died at his home in Greenville, Mich., about September 15, aged 86.

